# Attention bias modification: the Emperor's new suit?

**DOI:** 10.1186/1741-7015-10-63

**Published:** 2012-06-25

**Authors:** Paul MG Emmelkamp

**Affiliations:** 1 Department of Clinical Psychology, University of Amsterdam, Weesperplein 4, 1018 XA Amsterdam, The Netherlands

**Keywords:** Attention bias modification, demand characteristics, social anxiety disorder, social phobia

## Abstract

A series of primarily laboratory-based studies found attention bias modification in socially anxious participants to lead to reduced anxiety. It is argued that the failure to replicate the positive results of attention bias modification in the study of Carlbring *et al*. may be due to reasons other than the application through the Internet. A number of controlled studies failed to replicate the positive effects of attention bias modification in clinically rather than subclinically socially anxious subjects. Given the lack of robust evidence for attention bias modification in clinically socially anxious individuals, the author is inclined to consider attention bias modification as 'the Emperor's new suit'. Results achieved with regular Internet-based treatments for social anxiety disorder based on cognitive therapy and exposure methods are much better than those achieved with attention bias modification procedures delivered 'face to face' in clinically distressed participants. Given the lack of robust evidence for attention bias modification in clinical samples, there is no need yet to investigate the implementation of attention bias modification through the Internet.

Please see related article: http://www.biomedcentral.com/1471-244X/12/66

## Background

A growing number of studies have accumulated over the past 30 years that support the psychological treatment of social anxiety disorder [1,2]. In a meta-analysis of active treatments in clinically relevant samples, Powers *et al*. [2] found clear support for cognitive-behavioral treatments (CBTs). Contrary to what one would expect, cognitive therapy did not outperform exposure treatment; neither did cognitive therapy enhance the effects of exposure-based treatments. It is interesting to note that, while not significantly different, exposure methods produced the largest controlled effect size relative to cognitive or combined methods. Nevertheless, cognitive therapies are very popular among clinicians, as is research into cognitive mechanisms in social anxiety disorders. Although the emphasis was originally on explicit measures, more recent research in the domain of experimental psychopathology has increasingly focused on implicit processes involved in anxiety disorders, including attentional bias. A series of primarily laboratory-based studies found attention bias modification in socially anxious participants to lead to reduced anxiety [3]. In the randomized controlled trial (RCT) of Carlbring *et al*. [4], this procedure was applied through the Internet, but the positive effects of attention bias training found in previous studies could not be replicated in clinically socially anxious individuals. The authors of this RCT interpret the failure to replicate these positive results primarily in terms of problems associated with the delivery of attention training through the Internet. The aim of this paper is to discuss other potential reasons for these negative results and the current problems with the implementation of attention training in clinical samples.

### Clinical relevance of research into experimental psychopathology

Research into experimental psychopathology has grown exponentially over the last two decades, but -despite hundreds of studies published in journals such as *Behaviour Research and Therapy*, *Journal of Abnormal Psychology *and *Journal of Behavior Therapy and Experimental Psychiatry*, this type of research has resulted in hardly any clinical application. A large series of analogue studies in non-clinical samples has consistently shown that attention bias and anxiety are related [5]. Although this is usually interpreted as evidence for attentional bias being a vulnerability factor for developing anxiety, only few studies investigated this directly [6]. So far, the only promising clinical applications derived from research into experimental psychopathology are cognitive bias modification procedures. Cognitive bias modification procedures systematically train changes in patterns of selective attention and selective interpretation. Based on cognitive theories of social anxiety, which hold that socially anxious individuals selectively attend to social threat cues, it is assumed that changing these biases by attention bias modification will lead to positive changes in social anxiety. Over the course of many trials, participants are expected to implicitly learn to attend selectively to non-threatening stimuli rather than threatening stimuli. These studies showed that anxious individuals are no faster to respond to probes replacing threat cues than to non-threat cues, but they are slower to respond to probes that are opposite to threat cues relative to non-threat cues.

Although originally evaluated in analogue samples, typically consisting of paid undergraduate (psychology) students [3,7], a few studies have now evaluated the effects of attention bias modification in more clinically relevant socially anxious individuals, including the study of Carlbring *et al*. [4]. Although two RCTs found eight sessions of attention bias modification to be superior to a comparable placebo condition [8,9], results on clinician rating and self-report questionnaires in the Schmidt *et al*. study [9] were non-significant at post-treatment; results only became statistically significant at the four-month follow-up. Between-group effect sizes (Cohen's d) at follow-up (d = 0.35 to 0.41) were small. Although using the identical treatment protocol to Schmidt *et al*., Amir *et al*. [8] found much larger between-group differences (d = 0.69 to 1.59) than in the Schmidt *et al*. study [9]. These differences in outcome are difficult to explain, given that the same materials and procedures were used in the two studies. Additionally, in another recent RCT [10], there were no significant group × time interactions for the self-reported measures of anxiety. Further, engagement toward non-threat cues did not have any effect, only training to disengage from threat led to a small reduction in anxiety, but only on a behavioral measure. Other negative results of attention bias modification with clinically socially anxious individuals were reported in an RCT by Julian *et al*. [11]. These authors also used identical assessment and training procedures to those used by Amir *et al*. [8]. An RCT by McEvoy and Perini [12] using a different attention training task revealed that attention training did not enhance the effects of standard CBTs in clinically socially anxious individuals. Finally, theoretically, there is still no robust evidence that the cognitive change found is predicted by performance changes on a cognitive task measuring the cognitive process of interest [10].

Taken together, the results of the studies investigating attention training in clinically socially anxious individuals suggest there is no robust evidence that attention training is of clinical value. So far, only the study by Amir *et al*. [8] has produced clinically relevant results, which are difficult to interpret given the small or negative results of a series of other clinical studies [4,9-12].

### Do we need attention bias modification in internet-based treatment?

Given the lack of robust evidence for attention bias modification in clinical samples, there is no need yet to investigate the implementation of attention bias modification using the Internet. Internet-based treatments for social phobia have been intensively studied in the last few years [13]. Results reveal that Internet-based CBT treatments for social phobia are generally more effective than waiting list control. Face-to-face treatment for fear of public speaking has been shown to be as effective and accepted as the same treatment applied over the Internet without any contact with a therapist, but contact with the therapist during treatment increases treatment compliance and enhances treatment outcome. In a recent study by this author's group [14], an exposure-based Internet treatment 'Talk to me' was compared with a waiting list control group. The 'Talk to me' treatment was significantly more effective than the control treatment on a number of measures: fear and avoidance to the target behaviors, fear of public speaking, and work impairment. Regarding the effect size (Cohen's d) for the measures related to social phobia the Internet treatment had a high within-group (d = 1.13) and between-group (d = 0.86) effect size. The effect sizes achieved with the 'Talk to me' program are comparable to results of face-to-face treatment of social phobia in a recent meta-analysis by Powers *et al*. [2]. Thus, results achieved with regular Internet-based treatments for social anxiety based on cognitive therapy and exposure methods are much better than those achieved with attention bias modification procedures.

### Future research

If attention bias modification is indeed related to improvement of social anxiety, which has not yet been demonstrated convincingly, it would be very interesting to see whether the same applies to evidence-based psychological treatments. One study [15] found that successful CBT led to reduced attention bias in socially anxious individuals, but this study was published about 20 years ago. So there is a clear need for studies investigating the impact of successful cognitive therapy on attentional bias. The most effective treatment for social phobia is probably exposure *in vivo *[2]. Whether changes in attentional bias and interpretation bias are also achieved with pure exposure methods is theoretically very interesting. Are the patients who most improve with exposure also the ones who most normalize their attentional bias? Also, research is needed to establish whether patients who improve with cognitive and exposure methods, but who do not change the attention and interpretation biases, are more at risk for relapse than patients who improve and change these biases.

One of the factors involved in the improvements achieved with attention bias modification may be expectancy of therapeutic gain. One way of investigating the potential role of expectancy of therapeutic gain is by varying the instructions individuals receive. For example, half of the participants undergoing attention bias modification and control procedures may be informed that they will undergo a bonafide treatment for social anxiety, while the other half might be led to believe that they are participating in a study investigating threat cues and neutral cues on physiology. Such designs were popular in the 1970s, but may still be valuable to rule out the role played by demand characteristics in attention bias and interpretation training.

## Conclusions

Most of the evidence in favor of cognitive modification procedures is based on analogue research and few studies have investigated the effects in clinical samples. As noted by Beard [6], none of the studies followed the Consolidated Standards of Reporting Trials guidelines and most did not identify a primary outcome measure. To be able to objectively evaluate the potential benefits of these procedures, clinical trials have to be registered in clinical trial registers before the start of the study, as was done by Carlbring *et al*. [4]. By doing so, one knows how many studies have evaluated these approaches and how many of these studies have resulted in positive or negative outcome on the primary outcome measure. For example, to date, only one study [16] has found positive effects of cognitive bias modification in patients with generalized anxiety disorder, but close inspection of the results reveal that results were not evident on the worry scale, which is generally held to be the primary outcome measure in patients with generalized anxiety disorder.

It has been argued that there is a clear need for procedures like attention bias modification, since a number of individuals do not improve after having received evidence-based CBT treatment. As a clinician, this author would welcome research efforts into the additional value of attention bias modification for the failures of behavioral and CBTs. As long as these studies are lacking, however, this author is inclined to consider attention bias modification as 'the Emperor's new suit'.

## Competing interests

The author declares that he has no competing interests.

## Author's information

Since 2006, PMGE has been appointed as Academy Professor by the Royal Academy of Arts and Sciences. He is Co-Editor-in-Chief of *Clinical Psychology and Psychotherapy *and Section Editor of *BMC Psychiatry*.

## Pre-publication history

The pre-publication history for this paper can be accessed here:

http://www.biomedcentral.com/1741-7015/10/63/prepub
